# Do an invasive organism's dispersal characteristics affect how we should search for it?

**DOI:** 10.1098/rsos.171784

**Published:** 2018-03-21

**Authors:** Maggie D. Triska, Michael Renton

**Affiliations:** 1 Schools of Biological Sciences, Agriculture and Environment, The University of Western Australia, 35 Stirling Highway, Crawley, Western Australia 6009, Australia; 2 Plant Biosecurity Cooperative Research Centre, Level 2, Building 22, Innovation Centre, University Drive, University of Canberra, Bruce, ACT 2617, Australia

**Keywords:** biosecurity, diffusion, invasion biology, leptokurtic, pests, spatial spread

## Abstract

We investigated how an invading organism's dispersal characteristics affect the efficacy of different surveillance strategies aimed at detecting that organism as it spreads following a new incursion. Specifically, we assessed whether, out of the surveillance strategies tested, the best surveillance strategy for an organism varied depending on the way it disperses. We simulated the spread of invasive organisms with different dispersal characteristics including leptokurtic and non-leptokurtic kernels with different median dispersal distances and degrees of kurtosis. We evaluated surveillance strategies with different sampling arrangements, densities and frequencies. Surveillance outcomes compared included the time to detection, the total spread of the invasion and the likelihood of the invasion reaching new areas. Overall, dispersal characteristics affected the surveillance outcomes, but the grid surveillance arrangement consistently performed best in terms of early detection and reduced spread within and between fields. Additionally, the results suggest that dispersal characteristics may influence spread to new areas and surveillance strategies. Therefore, knowledge on an invasive organism's dispersal characteristics may influence how we search for it and how we manage the invasion to prevent spread to new areas.

## 1. Background

Biological invasions often have negative impacts on the environment and economy including reduced biodiversity, trade restrictions and high management costs [1]. Consequently, prevention is the best defence against biological invasions, but often legislation and quarantine procedures are not enough to restrict a new invasion from occurring [2]. When prevention fails, surveillance strategies are required to identify invasions quickly to alleviate the potential negative impacts of such invasions [3]. Surveillance strategies aim to improve the likelihood of identifying invasions early, thus increasing the probability of successful eradication efforts [1,4,5].

To evaluate surveillance strategies for the early detection of biological invasions, we must account for both their costs (e.g. the search effort) and their efficacy (e.g. the probability of detecting the organism within a certain time of its first arrival) [3]. The efficacy of a given surveillance strategy is unlikely to be the same for all organisms, and instead is expected to be dependent on the organism's biological characteristics and the way these interact with the environment [6,7]. Most organisms are likely to have some ability to spread by themselves; this is typically a density-dependent diffusion type of spread. Additionally, most invasive organisms exhibit occasional, rare long-distance dispersal events, usually resulting from human or environmental vectors, which travel a greater distance than the usual diffusion and have the potential to increase greatly the spread of an invasion [8–11]. The frequency and distance of these long-distance dispersal events, and the rate at which spread occurs at other times, will vary from one organism to another and strongly influence the way each organism spreads.

Surveillance strategies are typically designed using information from prior and current monitoring, reporting and predictive modelling [1,12]. However, current models often do not account for specific dispersal characteristics, due in part to the challenge of predicting rare, stochastic, long-distance dispersal events, and instead, they focus on the evaluation of surveillance strategies based on detection probability or resource availability [3,13–16]. Therefore, there is a need to test whether an organism's dispersal characteristics will affect its spread patterns in a way that impacts the efficacy of different surveillance strategies, and their relative efficiency (i.e. which strategy is most effective, for a given cost). If this is indeed the case, it will indicate a clear need to incorporate different dispersal characteristics, and particularly occasional long-distance dispersal, into spread models used to test surveillance strategies, even if they are hard to predict or quantify [9,17,18].

The aim of this study was to investigate whether the effectiveness of different surveillance strategies to detect new biological invasions depends on the organism's dispersal characteristics. In terms of dispersal characteristics, we were interested in median and mean dispersal distance, and the frequency and distance of occasional long-distance dispersal, which corresponds to the degree to which the organism's dispersal kernel is leptokurtic or ‘fat-tailed’. We wanted to assess effectiveness via a range of measures, including the time to detection, the maximum spread distance, the area infested at the time of detection and the amount of spread beyond the area under surveillance. These measures were selected as they can be linked to the likelihood of spread to new areas, the probability of eradication and the cost of that eradication. We also considered a variety of surveillance strategies, including different sampling arrangements, densities and frequencies.

## 2. Material and methods

We used a general spread model to simulate the spread of a new biological invasion. The stochastic model, implemented in R [19], simulates initial spread of an invasive organism across a landscape where some areas are suitable, and others are unsuitable. The model uses a simple function to represent population dynamics within each pixel. The function relates time since initial colonization to the number of propagules produced in the pixel. It depends on four model parameters: *p*_max_, the maximum number offspring per generation; *r*_0_, the rate of spread; *t*_0_, the time from first infestation/colonization to the start of reproduction; and *t*_50_, the time from first infestation/colonization to when 50% of the maximum reproduction is reached (table 1). Another model parameter *p*_estab_ specifies the proportion of offspring that establish upon reaching a suitable habitat. The model assumes passive isotropic dispersal, that is, the pest has equal chance of dispersing in any direction and does not actively search for any particular type of habitat or host. We simulated dispersal separately for each propagule and generated dispersal distance as a random draw from a dispersal kernel. The model is based on previous biological spread models [20,21]; for additional details of the model, see [6].

**Table 1.** The spread model parameters and outputs used in this study (explanations and units of outputs are in italics).

symbol	explanation	units, value/range
*p*_max_	number offspring/generation	10 000
*r*_0_	rate of spread	1.0 m year^−1^
*t*_0_	start of reproduction	5 months
*t*_50_	time at which 50% of the offspring maximum is reached	15 months
*p*_estab_	proportion of offspring that establish	0.01
*d*_50_	distance traversed by 50% of the pests	0.005–1.5 m
*d*_99_	distance traversed by 99% of the pests	5 or 10 m
*p*_detect_	likelihood that the pest is detected if present	0.01, 0.25, 0.75 1.00
*T*	*time to detection*	*0–400 months*
*Max*_dist_	*maximum distance travelled at time T*	*0–300 m*
*No*_fields_	*number of fields infested at time T*	*1–25 fields*
*Area*	*area infested (1 × 1 m pixels) at time T*	*0–250 000 m^2^*
*E*	*number of escapes*	*0–Inf*

**Figure 1.**
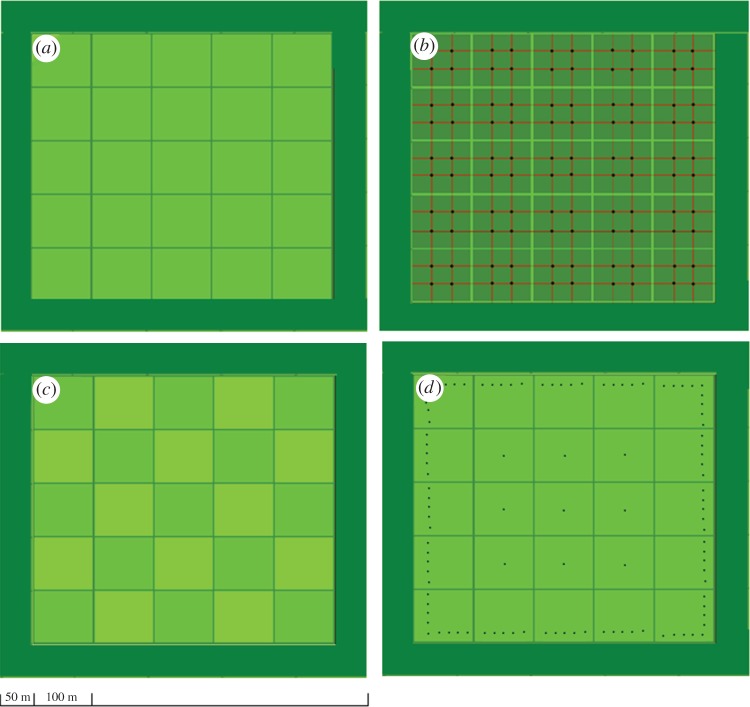
The landscape over which pest spread was simulated; it represents a total area of 614 × 614 m, individual fields are 100 × 100 m and the surrounding buffer is 50 m wide (*a*). The surveillance design included sample points being located randomly, in a grid or in a strategic arrangement. In (*b*), the black dots indicate a surveillance density of 4 points/field in a grid arrangement. The lowest surveillance density modelled was 1 point/2 fields, with the field in which the point was located being alternated between model runs (*c*) so that the sample points were not always located in the same field (i.e. in run one surveillance occurred in the dark green fields and in run two surveillance occurred in the light green fields). The strategic placement focused surveillance efforts along the border of the farm, with only 1 point located in each field not on the border (*d*). Different arrangements were considered with the same total number of sampling points (e.g. the grid arrangement in (*b*) has the same number of points as the strategic border arrangement in (*d*)).

For this study, all simulations were conducted on the same landscape, which could represent, for example, a horticultural farm, an orchard, a tree plantation or remnants of natural vegetation separated by roads (figure 1). For simplicity, we describe it as a farm where the simulated landscape consists of 1 × 1 m square pixels (total square area of 614 × 614 m; 37.70 ha). The farm is subdivided into 25 blocks of suitable habitat or ‘fields’, each 100 × 100 m (1.00 ha), which are separated by 2 m divisions that represent plants or roads, and there is a 50 m buffer around the fields (figure 1*a*). The pest cannot establish in the divisions between fields or in the buffer, reflecting an assumption that conditions are unsuitable or a suitable host is not present.

We conducted a series of spread simulations with all model parameters, except for dispersal, held at fixed values (table 1). We used two non-leptokurtic (exponential) and three leptokurtic (Weibull) dispersal kernels (table 2). For each kernel, we conducted 100 replicate simulation runs, with each run simulating spread for 200–400 time steps (nominally representing months; see table 1 for variable input values). In each simulation run, the initial location of the invasion was randomly selected within one of the 25 fields, and then spread from this initial site was simulated.

**Table 2.** The *d*_50_ and *d*_99_ values used in each dispersal kernel to simulate dispersal and spread. Dispersal kernel formulae: exponential, *f*(*x*) = *λ*e^*−λx*^; and Weibull, *f*(*x*) = *kλx^*k*−1^*exp(−*λx^*k*^*).

abbreviation	dispersal kernel	*d*_50_	*d*_99_	*k*	*λ*
Exp	exponential	$5\left(\frac{\text{log}(0.5)}{\text{log}(0.01)}\right)$	5	^a^	1.0857
Exp^+^	exponential	$10\left(\frac{\text{log}(0.5)}{\text{log}(0.01)}\right)$	10	^a^	2.1715
Wei	Weibull	0.05	5	0.4112	0.1219
Wei^−^	Weibull	0.025	2.5	0.4112	0.0610
Wei^+^	Weibull	0.0005	5	0.2056	0.0030

^a^The scale parameter (*k*) is not relevant for the exponential kernels.

The five dispersal kernels were chosen to enable comparison between different dispersal distances, and also different levels of kurtosis (fat-tailedness). Each kernel could be described by two parameters: *d*_50_, the median dispersal distance, or the distance that half the propagules exceed; and *d*_99_, the 99th percentile dispersal distance, or the distance that only 1% of propagules exceed (table 1). The exponential kernel is defined by one parameter, and so specifying either one *d*_99_ or *d*_50_ will determine the other (and also the distribution scale parameter *k*). The Weibull kernel is defined by two parameters, and so specifying both the *d*_50_ and *d*_99_ values will determine the scale (*k*) and shape (*λ*) parameters of the distribution (table 2). Our first exponential kernel (Exp) used the same *d*_99_ value as the first Weibull kernel (Wei) and the second exponential kernel (Exp^+^) had a greater *d*_99_ value to more closely mimic the spread of the Weibull distributions. The second Weibull kernel (Wei^−^) had both *d*_50_ and *d*_99_ decreased by a factor of two, compared with the first (Wei), meaning that it was equally as leptokurtic, but just generated smaller distances. The two exponential kernels could thus be described as non-leptokurtic, and the first two Weibull kernels as mediumly leptokurtic. The third Weibull kernel (Wei^+^) was used to represent an organism with extremely leptokurtic dispersal; this one had the same *d*_99_ as the first (Wei) but a much smaller *d*_50_.

**Figure 2.**
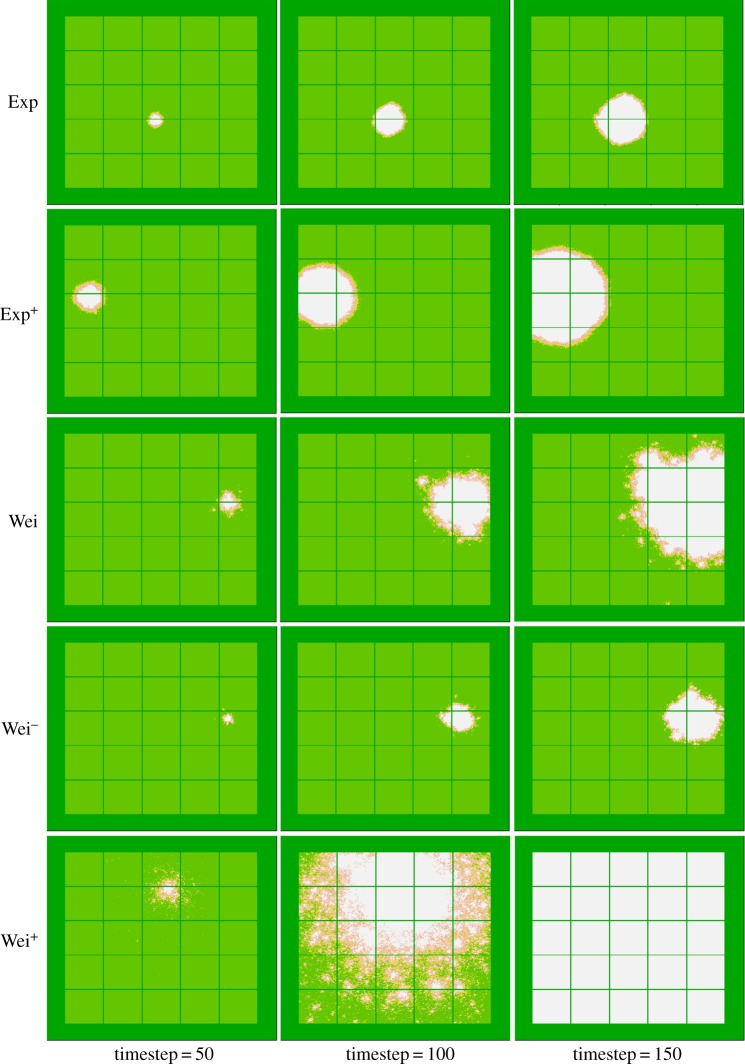
Example simulated spread output for each of our five (Exp, Exp^+^, Wei, Wei^−^ and Wei^+^) dispersal kernels at timesteps 50, 100 and 150.

The five kernels result in distinct patterns of spread (figure 2). The non-leptokurtic exponential kernels result in diffusion-style spread, with a constant rate of linear expansion, a relatively smooth invasion front, and no satellite infestations appearing beyond the invasion front. The leptokurtic Weibull kernels and particularly Wei^+^ result in typical leptokurtic spread, with a rate of expansion that increases with time (exponential expansion), a relatively unsmooth invasion front, and clear satellite infestations appearing beyond the invasion front. As desired, in the early stages of spread, the Wei^−^ kernel results in similar spread area to the Exp kernel and the Exp^+^ kernel results in similar spread area to the Wei kernel. The Wei^+^ kernel results in similar spread area in the earliest stages as well, but quickly overtakes the others. The fat-tail of the leptokurtic kernels resulted in individuals escaping the simulated area (i.e. jumping beyond the 50 m buffer around the fields) and so the number of such escapes at each timestep was recorded during each simulation.

Next, we simulated the process of detection under different surveillance strategies. In this study, a surveillance strategy is defined by the spatial locations or points at which sampling occurs, with different strategies having different sampling densities, frequencies and/or arrangements. ‘Sampling points’ is used in a general sense, and could refer to locations at which soil or leaf samples are taken, or the locations at which traps are placed, etc. Surveillance *arrangement* was based on standard sampling designs, including grid, random and strategic arrangements. In the grid arrangement, sampling always occurred at the same points on a regular grid (figure 1*b*). In the random arrangement, the number of points per field remained constant, but the exact locations within each field were randomly selected at the start of each simulation. In the strategic arrangement the same number of points as in the grid and random were used, but the placement focused on the edges of the farm, having just one point in the middle of each field and all additional sampling points located near the border of the simulated area (figure 1*d*). Surveillance density and frequency were also varied. Surveillance *density* ranged from 1 sampling point per two fields (0.5 points/field; alternating field location between runs (figure 1*c*)) to 1, 2, 4, 9, 16 or 25 points/field. Surveillance *frequency* ranged from sampling once per month to once every 2, 3, 4, 6, 8 or 12 months. For all strategies, we also considered how detection probability affected the surveillance efficacy. Four detection probabilities (*p*_detect_) were assessed: perfect detection probability (1.00) and three imperfect detection probabilities, very low (0.01), low (0.25) and high (0.75). Every simulated spread output, for each replicate with each dispersal kernel, was checked against each combination of surveillance strategy and detection probability, and the first timestep at which a detection occurred was recorded. Detection was assumed to occur with a probability, *p*_detect_, in any pixel that was infested and where sampling occurred at that time. At the timestep when detection first occurred, multiple output variables were calculated and recorded, including: the time to detection (*t*), the area infested (*Area*), the maximum distance of infestation from the initial incursion point (*Max*_dist_), the number of fields infested (*No*_Fields_) and the number of escapes, or propagules that went beyond the 50 m buffer (*E*) (table 1). These four output variables are assumed to be closely related to the probability of being able to eradicate the species following detection, the cost of such eradication and the probability of the species spreading onwards to other fields. Basic statistics were calculated for each of these output variables across the 100 replicate runs, for every combination of dispersal kernel (Exp, Exp^+^, Wei, Wei^−^, Wei^+^), arrangement (grid, random and strategic), density (number of points per field: 0.5, 1, 2, 4, 9, 16 or 25), frequency (1, 2, 3, 4, 6, 8 or 12) and detection probability (0.01, 0.25, 0.75 and 1.00). Full model and surveillance code can be accessed from the University of Western Australia's online repository [22].

## 3. Results

### 3.1. Overview

**Figure 3.**
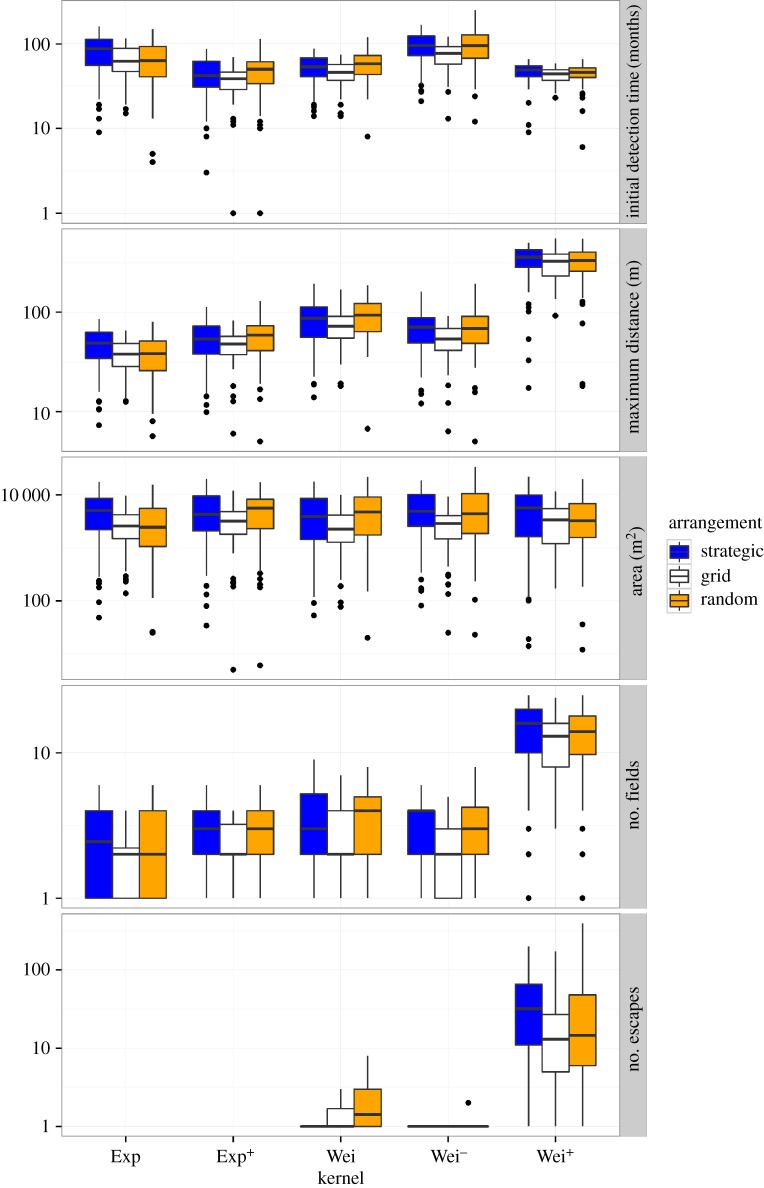
Summary of five output variables (top to bottom panels) from 100 replicate runs for five different dispersal kernels and three different surveillance arrangements, for a detection probability of 75%, a surveillance density of two and a surveillance frequency of one. Box plots indicate the median of the 100 replicate runs (internal line), the interquartile range (box) and the range (whiskers), with any outliers (results more than 1.5 times the interquartile range from the median) shown as individual points. Initial detection time is the number of months until the first detection occurs. Maximum distance represents the maximum distance in metres travelled by the pest at the time of detection. The suitable area infested is the number of 1 × 1 m pixels that were infested at the time of detection (maximum suitable pixels = 250 000). The number of fields represents the number of fields with any infestation at the time of detection (maximum possible fields = 25). Finally, the number of escapes indicates how many seed, or propagules, had travelled beyond the 50 m border at the time of detection. Note log-scale on *y*-axis.

**Figure 4.**
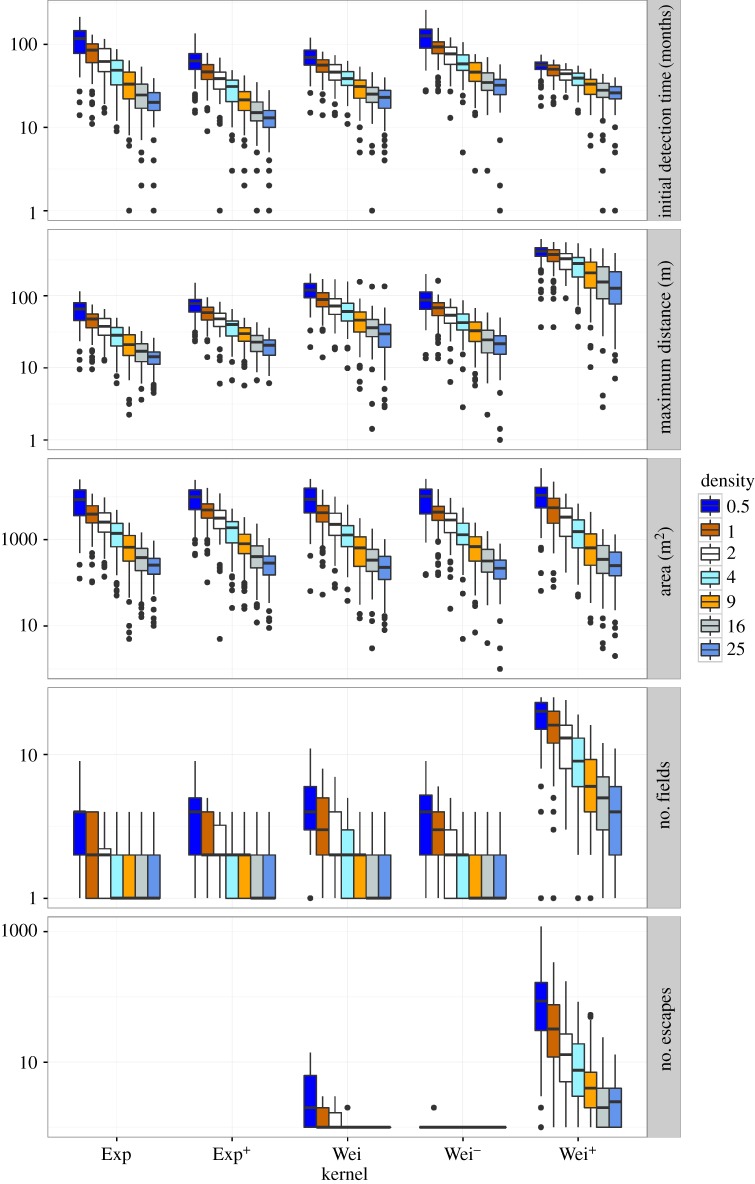
Summary of five output variables (top to bottom panels) from 100 replicate runs for five different dispersal kernels and seven different surveillance densities, for a detection probability of 75%, a grid arrangement and a surveillance frequency of one. Box plots indicate the median of the 100 replicate runs (internal line), the interquartile range (box) and the range (whiskers), with any outliers (results more than 1.5 times the interquartile range from the median) shown as individual points. Initial detection time is the number of months until the first detection occurs. Maximum distance represents the maximum distance in metres travelled by the pest at the time of detection. The suitable area infested is the number of 1 × 1 m pixels that were infested at the time of detection (maximum suitable pixels = 250 000). The number of fields represents the number of fields with any infestation at the time of detection (maximum possible fields = 25). Finally, the number of escapes indicates how many seed, or propagules, had travelled beyond the 50 m border at the time of detection. Note log-scale on *y*-axis.

**Figure 5.**
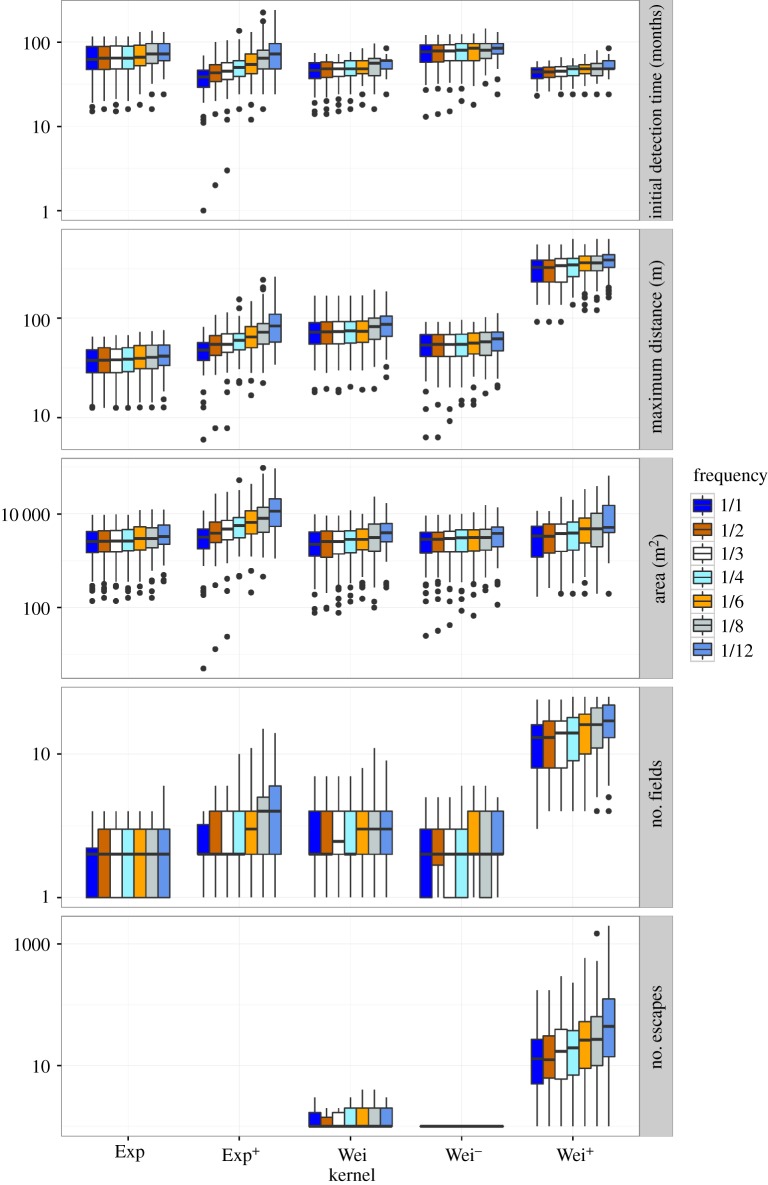
Summary of five output variables (top to bottom panels) from 100 replicate runs for five different dispersal kernels and 12 different surveillance frequencies (number of months per year; i.e. 1/1 means monthly surveillance, 1/3 means quarterly surveillance and 1/12 means yearly surveillance), for a detection probability of 75%, a grid arrangement and a surveillance density of 2. Box plots indicate the median of the 100 replicate runs (internal line), the interquartile range (box), and the range (whiskers), with any outliers (results more than 1.5 times the interquartile range from the median) shown as individual points. Initial detection time is the number of months until detection occurs. Maximum distance represents the maximum distance in metres travelled by the pest at the time of detection. The area infested is the number of 1 × 1 m pixels that were infested at the time of detection (maximum suitable pixels = 250 000). The number of fields represents the number of fields with any infestation at the time of detection (maximum possible fields = 25). Finally, the number of escapes indicates how many seed, or propagules, had travelled beyond the 50 m border at the time of detection. Note log-scale on *y*-axis.

Different dispersal kernels resulted in different surveillance outcomes, regardless of surveillance density, frequency, arrangement or detection probability (figures 3–5; electronic supplementary material, appendix S1). The dispersal kernel giving the worst (or best) result under one surveillance measure was not necessarily the same kernel that gave the worst (or best) result under another surveillance measure. For example, at low surveillance density, Wei^+^ resulted in the highest maximum distance, area infested and number of fields, but the lowest time to detection (figure 4). Even more interestingly, which surveillance arrangement type was best out of the strategies simulated depended on both the measure of surveillance efficacy and the dispersal kernel in combination (figure 3). Specifically, the grid arrangement gave fewer escapes than the strategic border arrangement for the most leptokurtic kernel (Wei^+^), whereas the strategic border arrangement gave fewer escapes for the Wei kernel (figure 3; electronic supplementary material, appendix S2). On the other hand, the grid arrangement outperformed the strategic border arrangement for the Wei and Wei^+^ kernels when the surveillance measure was time to detection (figure 3).

Surveillance density (figure 4) and surveillance frequency (figure 5) affected measures of surveillance efficacy in the expected ways, although there were still interesting interactions with dispersal kernel. For example, increasing surveillance density caused a greater reduction in time to detection for the less leptokurtic kernels (e.g. Exp) than for the more leptokurtic (e.g. Wei^+^) kernels (figure 4). As detection probability decreased, time to detection and spread at detection increased, as would be expected, and the variability in outputs between replicate runs also increased (electronic supplementary material, appendix S1). At the lowest detection probability (0.01), infestations were not always detected (kernel (% not detected): Exp (14%), Exp^+^ (7%), Wei (16%), Wei^−^ (22%) and Wei^+^ (23%)) but at all other detection probabilities infestations were always detected for all kernels. The effects of varying density, frequency, detection probability and arrangement were largely independent, and so we only attempt to show interactions between each of these and dispersal kernel (figures 3–5 and electronic supplementary material, appendix S1). We present median values and ranges of results in figures and the mean ± s.e. are presented in text when appropriate.

### 3.2. Surveillance arrangement

Surveillance arrangement affected the time to detection and spread for all dispersal kernels, with the grid arrangement generally giving the quickest time to detection and strategic and random arrangements the slowest (figure 3). The grid arrangement also generally resulted in the lowest within-field spread (including maximum distance, area and number of fields). The grid arrangement had the lowest number of escapes for the highly leptokurtic Wei^+^ dispersal kernel (figure 3; electronic supplementary material, appendix S2). By contrast, for the less leptokurtic Wei dispersal kernel, the strategic and grid arrangements resulted in similar number of escapes, with the strategic arrangement sometimes performing best. For the Exp dispersal kernels, the number of escapes was zero for all surveillance arrangements.

### 3.3. Surveillance density

Overall, the time to detection for all dispersal kernels decreased as the density of samples increased (figure 4). Increasing surveillance density caused a greater reduction in time to detection for the less leptokurtic kernels (e.g. Exp) than for the more leptokurtic (e.g. Wei^+^) kernels. The time to initial detection was highest in the Wei^−^ kernel across all sampling densities (123.50 ± 4.17 months at 0.5 points/field), which decreased as the surveillance density increased to 31.08 months ± 1.12 months at 25 points/field.

The maximum distance travelled by the invasion decreased as surveillance density increased for all dispersal kernels, and at similar rates for all kernels (figure 4). The maximum distance travelled for the Wei, Wei^−^ and Exp^+^ kernels always occurred between the maximum distance travelled for Wei^+^ (highest) and Exp (lowest) kernels regardless of the surveillance density. For the lowest surveillance density (0.5 points/field) the maximum distance travelled was highest for the Wei^+^ kernel (397.59 ± 10.19 m) and lowest for the Exp kernel (63.63 ± 2.46 m), with the invasion travelling further for the Wei kernel, than for the Wei^−^ and Exp^+^ kernels (119.38 ± 3.95 m, 89.80 ± 3.29 m and 74.83 ± 2.32 m, respectively).

The area infested (m^2^) at the time of detection decreased as the surveillance density increased (figure 4). The percent of the suitable landscape infested was highest when the surveillance density was lowest for all dispersal kernels, Wei^+^ (12 065.90 ± 905.32 m^2^, 4.83% of the total field area), Wei^−^ (10 167.22 ± 655.12 m^2^, 4.07% of the total field area), Wei (10 105.65 ± 674.63 m^2^, 4.04% of the total field area), Exp^+^ (10 051.17 ± 586.05 m^2^, 4.02% of the total field area) and Exp (9333.93 ± 631.44 m^2^, 3.73% of the total field area). The total area infested decreased to less than 1% in all dispersal kernels as the surveillance density increased to 4 or more points/field.

The number of fields infested decreased for all dispersal kernels as surveillance density increased (figure 4). The greatest number of fields infested occurred with the Wei^+^ kernel and a total of 5.51 ± 0.55 infested fields occurred at the lowest survey density. The invasion did not travel as far under the other surveillance densities and 2.24 ± 0.22, 1.75 ± 0.17, 1.91 ± 0.19, and 1.89 ± 0.19 fields were infested for Wei, Wei-, Exp and Exp+ kernels, respectively.

The number of escapes decreased as survey density increased (figure 4; electronic supplementary material, appendix S2). Escapes only occurred for the leptokurtic Weibull dispersal kernels, with no escapes for the exponential kernels at any surveillance density. The probability and expected number of escapes for the Wei^−^ kernel were dependent on the surveillance density, with few escapes (range 0.01–0.07) occurring when ≤2 points/field were simulated and zero escapes when ≥4 points/field were simulated (electronic supplementary material, appendix S2). The highest numbers of escapes were recorded for the Wei^+^ kernel at all surveillance densities with 140.43 ± 20.36 escapes occurring at the lowest density and 1.33 ± 0.22 escapes occurring at the highest density for this kernel.

### 3.4. Surveillance frequency

The mean time to detection increased with decreasing surveillance frequency for all dispersal kernels (figure 5). However, this effect of surveillance frequency was small: as surveillance frequency varied from the maximum (monthly) to the minimum (yearly), the time to detection varied from 75.85 ± 2.30 to 84.84 ± 2.43 months for the Wei^−^ kernel, and from 37.87 ± 1.24 to 47.28 ± 1.41 months for the Exp^+^ kernel. The effect of surveillance frequency on time to detection was greatest for the Exp^+^ kernel and smaller for the more leptokurtic kernels.

Surveillance frequency had a similarly small effect on maximum distance travelled by the invasion, area infested (m^2^) and number of fields infested at the time of detection, with only small improvements observed with more frequent sampling (figure 5). At all surveillance frequencies, both maximum distance and number of fields were greatest for the Wei^+^ kernel and smallest for the Exp kernel. Infested area tended to be greatest for the Exp^+^ kernel but there was little difference between the kernels over the range of surveillance frequencies. The effect of surveillance frequency on all three of these within-field spread measures (distance, area, fields) was greatest for the Exp^+^ and Wei^+^ kernels. The number of escapes also decreased with increased frequency and the largest impact occurred for the Wei^+^ kernel for which increased survey density from yearly to monthly reduced the number of escapes from 120.87 ± 24.19 to 23.70 ± 3.33.

## 4. Discussion

The results of this study demonstrate that it is important to consider dispersal characteristics when designing surveillance strategies aimed at reducing the negative impacts of biological invasions through early detection. An invasive organism's dispersal characteristics affect how we should search for it. Expectedly, highly leptokurtic dispersal kernels spread faster than low or non-leptokurtic dispersal kernels and the highly leptokurtic kernels have a higher potential to infest new areas. This increase in invasion potential is driven by the isolated satellite/outpost loci of the invasion that long-distance dispersal creates which exponentially increases the rate of expansion [23]. Our results also showed organisms with highly leptokurtic dispersal were generally detected earlier than those with less or non-leptokurtic kernels, but their invasion extent at the time of initial detection (i.e. maximum distance travelled, area and number of fields infested) was greater.

Therefore, for an organism that displays leptokurtic dispersal we might consider a high surveillance density to maximize the likelihood of detection as quickly as possible before spread becomes too great, although for any particular pest and situation an economic analysis would be required to determine whether the benefits gained by earlier detection would be great enough to warrant the additional costs of the higher density. Additionally, the idea of ‘early detection’ may be misleading for an organism with highly leptokurtic dispersal, as a given surveillance strategy may detect it quicker than a second organism with less-leptokurtic dispersal, but despite this, the first organism may have spread further and across greater area than the second organism when the first detection occurs. Moreover, the organism with highly leptokurtic dispersal will probably have a greater likelihood of escaping from beyond the surveillance area, even if it is detected considerably earlier. In other words, relatively early detection may not be good enough for an organism with highly leptokurtic dispersal, and even earlier detection may be required. In contrast, early detection may not be very important for an organism with a slow spreading non-leptokurtic dispersal kernel, as a low-density, low-frequency surveillance strategy that detects the invasion relatively late is still likely to detect it before it has spread far or wide, and particularly before it has escaped the surveillance area. We can conclude that for a species exhibiting non-leptokurtic dispersal, a low surveillance density may be sufficient, for a species exhibiting leptokurtic dispersal, intense sampling may be required to make surveillance effective, and for a species exhibiting highly leptokurtic dispersal, there may be no effective surveillance strategy, or at least none that is feasible or economical. However, the model used in this study is intentionally generic, appropriate to our aim of finding general insights related to surveillance and dispersal characteristics. To determine which surveillance density and arrangement is best for any particular organism would require an economic analysis of the trade-off between surveillance costs and likely eradication and damage costs.

A more effective arrangement using the same surveillance density and frequency is likely to be of significant interest to biosecurity managers, because it achieves better outcomes with the same resources. Our results suggest that the grid arrangement was generally better than the alternative random or strategic arrangements, across most measures of surveillance efficacy, sampling densities and frequencies and dispersal kernels. However, there was one potentially important exception. The number of escapes from the surveillance area was at least as good with a strategic border-protecting arrangement as with a grid arrangement for the Wei kernel, even though it was higher for the Wei^+^ kernel. For the Exp kernels, the number of escapes was always zero, but this was only because we had a buffer region. Without the buffer region, we would expect the strategic border-protecting arrangement to give fewer escapes than a grid or random arrangement for the Exp kernels as well. This indicates that for non-leptokurtic or less-leptokurtic dispersal, a strategic border-protecting arrangement may perform as well as or even better than other more standard arrangements in terms of stopping spread beyond the surveillance area, which should be a key surveillance strategy evaluation criterion. Only for highly leptokurtic dispersal would grid arrangements be expected to give better outcomes. We can conclude that if stopping spread beyond the surveillance area is the most important objective, surveillance arrangement should be considered in light of an organism's dispersal characteristics.

We determined that regardless of the dispersal kernel increasing surveillance density had much greater impact on improving outcomes than did increasing frequency. This suggests that resources should be allocated to increasing density rather than frequency, unless perhaps the cost of increasing frequency is much lower. Equivalently, if costs are to be cut, it may be better to reduce frequency rather than reduce density. We also found that increasing density (and frequency) had more effect on organisms with non-leptokurtic dispersal than those with leptokurtic dispersal, which is unfortunate because organisms with leptokurtic dispersal are precisely those that need more effective surveillance, as explained above.

This study suggests that to best optimize or predict the effectiveness of surveillance we must identify if a species exhibits leptokurtic dispersal, and even the degree to which its dispersal is leptokurtic. Our results also suggest that effective surveillance is likely to be much more difficult and costly for organisms with more leptokurtic dispersal. We know that most species can display long-distance, leptokurtic dispersal, and those that cannot move far on their own often rely on vectors for long-distance dispersal [8–11]. Additionally, species and environments may have characteristics that increase the likelihood of vector and long-distance dispersal [24]. For invasive species, some of these characteristics include attachment (sticky or barbed appendages), aerodynamic properties, buoyancy and location (distance to roadways, high fragmentation and transportation hubs) [24,25]. Based on species and environmental characteristics, the potential vectors (wind, water, humans, etc.) of species can be defined. Management may then be used to reduce species movement or control the potential vectors and decrease their likelihood of causing long-distance dispersal events [26,27]. Alternatively, if the vectors cannot be reduced or controlled the spread of the invasion may be decreased through other strategies (i.e. crop rotation, planting resistant varieties, using hygiene procedures, etc.) [28]. Even better, if leptokurtic dispersal of an invasive organism seems likely to occur and difficult to manage, then no surveillance or eradication strategy may be cost-effective, and resources may be best allocated to preventing the initial spread and incursion occurring in the first place [29,30].

Our results demonstrate that the relationship between time until first detection and extent of spread at time of first detection may depend on an organism's dispersal characteristics. Therefore, species with low or non-leptokurtic dispersal may be candidates for eradication, but leptokurtic species may not be candidates for eradication due to their increased spread. This is supported by the fact that a small total area infested (i.e. one small, isolated infestation) has been linked to successful eradication efforts in multiple taxa (animalia, bacteria, fungi, plantae and viruses) and regions (Europe, Americas and Australasia) [4]. Therefore, to detect species before they have spread beyond the option of eradication, we must optimize surveillance. Our results suggest that increased surveillance density in a grid pattern may be the most successful strategy for quickly detecting organisms with leptokurtic dispersal, but strategic border-protecting arrangements may be better for stopping escapes. This supports other studies' findings that increased surveillance density along with higher species abundance [31] or surveillance arrangements that systematically cover the whole sampling area (i.e. grid or zigzag arrangements) may increase the likelihood of species detection [32,33]. However, our results also highlight that species detection may not be optimized with the same strategies as those that minimize escapes, and indeed it is the latter that may be more important in a biosecurity context.

Additionally, the results of this study may have implications beyond surveillance for invasive organisms. Surveillance models that incorporate dispersal characteristics do not only provide predictions for biological invasions in agricultural and natural ecosystems, but the general model and methodology may have implications for conservation, restoration and the persistence of species under climate change [34–36]. Although the aims of these endeavours may be conflicting (i.e. species control and eradication versus species conservation and establishment) they all depend on the ability to predict species’ spread and subsequent detection. We suggest that to estimate species' spread and design surveillance, it is likely essential to understand the species’ dispersal potential and characteristics. Whether for purposes of preventing invasions or for conservation or restoration, surveillance of species in new areas can be challenging as species are often patchy within the landscape, rare or small [37]. Results of this study and the modelling approach employed could be used to help determine how to detect rare, uncommon species by targeting their known habitat and their likely spread based on species' dispersal potentials, whether the aim is for conservation or eradication.

Our aim was to produce a general spread model that did not explicitly account for biological processes such as fecundity, mortality and time to maturity that may influence spread [38], in order to keep the model relatively simple and focused on dispersal characteristics. Additionally, the model does not incorporate spatial heterogeneity which likely impacts the spread rate of invasive species [7,39]. In fact, research suggests that improvements to the surveillance of invasive species may occur through the identification of suitable habitat and economic costs [13,40,41]. Therefore, the next steps of this research are to apply the general spread model to specific case studies and incorporate simple biological processes and the spatial heterogeneity of the landscape to determine if these characteristics, along with different dispersal types, influence surveillance strategies that include economic analyses.

## Supplementary Material

Detection probability output

## Supplementary Material

Weibull dispersal number of escapes

## Data accessibility

General spread model and surveillance code can be accessed from the University of Western Australia's online repository (http://dx.doi.org/10.4225/23/59f8279a6b418) [22].

## References

[1] Hulme PE 2014 An introduction to plant biosecurity: past, present and future. In The handbook of plant biosecurity (eds Gordh G, McKirdy S), pp. 1–25. Dordrecht, The Netherlands: Springer (doi:10.1007/978-94-007-7365-3_1)

[2] Simberloff D et al. 2013 Impacts of biological invasions: what's what and the way forward. Trends Ecol. Evol. 28, 58–66. (doi:10.1016/j.tree.2012.07.013) 22889499 10.1016/j.tree.2012.07.013

[3] Mehta SV, Haight RG, Homans FR, Polasky S, Venette RC 2007 Optimal detection and control strategies for invasive species management. Ecol. Econ. 61, 237–245. (doi:10.1016/j.ecolecon.2006.10.024)

[4] Pluess T, Cannon R, Jarošík V, Pergl J, Pyšek P, Bacher S 2012 When are eradication campaigns successful? A test of common assumptions. Biol. Invas. 14, 1365–1378. (doi:10.1007/s10530-011-0160-2)

[5] Rejmánek M, Pitcairn MJ 2002 When is eradication of exotic pest plants a realistic goal. In Turning the tide: the eradication of invasive species (eds Veitch CR, Clout MN), pp. 249–253. Gland and Cambridge, UK: IUCN SSC Invasive Species Specialist Group.

[6] Triska MD, Renton M 2015 A general model to simulate how an invading organism's dispersal characteristics influence its spread, and the implications for surveillance strategies. In MODSIM2015, 21st Int. Congress on Modelling and Simulation (eds Weber T, McPhee MJ, Anderssen RS), pp. 1268–1274. Gold Coast, Australia: Modelling and Simulation Society of Australia and New Zealand.

[7] Pitt JPW, Worner SP, Suarez AV 2009 Predicting argentine ant spread over the heterogeneous landscape using a spatially explicit stochastic model. Ecol. Appl. 19, 1176–1186. (doi:10.1890/08-1777.1) 19688925 10.1890/08-1777.1

[8] Kot M, Lewis MA, van den Driessche P 1996 Dispersal data and the spread of invading organisms. Ecology. 77, 2027–2042. (doi:10.2307/2265698)

[9] Hastings A et al. 2005 The spatial spread of invasions: new developments in theory and evidence. Ecol. Lett. 8, 91–101. (doi:10.1111/j.1461-0248.2004.00687.x)

[10] Nathan R, Klein E, Robledo-Arnuncio JJ, Revilla E 2012 Dispersal kernels: review. In Dispersal ecology and evolution (eds Colobert J, Baguette M, Benton TG, Bullock JM), pp. 186–210. Oxford, UK: Oxford University Press.

[11] Nathan R 2006 Long-distance dispersal of plants. Science 313, 786–788. (doi:10.1126/science.1124975) 16902126 10.1126/science.1124975

[12] Kalaris T et al. 2014 The role of surveillance methods and technologies in plant biosecurity. In The handbook of plant biosecurity (eds Gordh G, McKirdy S), pp. 309–337. Dordrecht, The Netherlands: Springer (doi:10.1007/978-94-007-7365-3_11)

[13] Berec L, Kean JM, Epanchin-Niell R, Liebhold AM, Haight RG 2015 Designing efficient surveys: spatial arrangement of sample points for detection of invasive species. Biol. Invas. 17, 445–459. (doi:10.1007/s10530-014-0742-x)

[14] Epanchin-Niell RS, Brockerhoff EG, Kean JM, Turner J 2014 Designing cost-efficient surveillance for early detection and control of multiple biological invaders. Ecol. Appl. 24, 1258–1274. (doi:10.1890/13-1331.1) 29160647 10.1890/13-1331.1

[15] Cacho OJ, Spring D, Hester S, Mac Nally R 2010 Allocating surveillance effort in the management of invasive species: a spatially-explicit model. Environ. Model. Softw. 25, 444–454. (doi:10.1016/j.envsoft.2009.10.014)

[16] Hester S, Cacho O 2012 Optimization of search strategies in managing biological invasions: a simulation approach. Human Ecol. Risk Assess. Int. J. 18, 181–199. (doi:10.1080/10807039.2012.632307)

[17] Higgins SI, Richardson DM 1999 Predicting plant migration rates in a changing world: the role of long-distance dispersal. Am. Nat. 153, 464–475. (doi:10.2307/2463662) 29578791 10.1086/303193

[18] Suarez AV, Holway DA, Case TJ 2001 Patterns of spread in biological invasions dominated by long-distance jump dispersal: insights from Argentine ants. Proc. Natl Acad. Sci. USA 98, 1095–1100. (doi:10.1073.pnas.98.3.1095) 11158600 10.1073/pnas.98.3.1095 PMC14714

[19] R Core Team. 2016 R: a language and environment for statistical computing. Vienna, Austria: R Foundation for Statistical Computing.

[20] Renton M, Savage D 2015 Statistical emulators of simulation models to inform surveillance and response to new biological invasions. In Biosecurity surveillance: quantitative approaches (eds Jarrad F, Low-Choy S, Mengersen K), pp. 296–312. Wallingford, UK: CABI Publishing.

[21] Savage D, Renton M 2014 Requirements design and implementation of a general model of biological invasion. Ecol. Modell. 272, 394–409. (doi:10.1016/j.ecolmodel.2013.10.001)

[22] Triska M, Renton M 2017 General spread model Perth, Australia: The University of Western Australia (doi:10.4225/23/59f8279a6b418)

[23] Hengeveld R 1989 Dynamics of biological invasions. London, UK: Springer.

[24] Davies KW, Sheley RL 2007 A conceptual framework for preventing the spatial dispersal of invasive plants. Weed Sci. 55, 178–184. (doi:10.1614/WS-07-067.1)

[25] Sakai AK et al. 2001 The population biology of invasive species. Ann. Rev. Ecol. Syst. 32, 305–332. (doi:10.1146/annurev.ecolsys.32.081501.114037)

[26] Sheley RL, Petroff JK 1999 Biology and management of noxious rangeland weeds. Corvallis, OR: Oregon State University Press.

[27] Goodell K, Parker IM, Gilbert GS 2000 Biological impacts of species invasions: implications for policy makers. In Incorporating science, economics, and sociology in developing sanitary and phytosanitary standards in international trade, pp. 87–117. Washington, DC: National Academy Press.

[28] Matyjaszczyk E 2015 Prevention methods for pest control and their use in Poland. Pest Manag. Sci. 71, 485–491. (doi:10.1002/ps.3795) 24729297 10.1002/ps.3795

[29] Myers JH, Simberloff D, Kuris AM, Carey JR 2000 Eradication revisited: dealing with exotic species. Trends Ecol. Evol. 15, 316–320. (doi:10.1016/S0169-5347(00)01914-5) 10884695 10.1016/s0169-5347(00)01914-5

[30] Leung B, Lodge DM, Finnoff D, Shogren JF, Lewis MA, Lamberti G 2002 An ounce of prevention or a pound of cure: bioeconomic risk analysis of invasive species. Proc. R. Soc. Lond. B 269, 2407–2413. (doi:10.1098/rsb.2002.2179) 10.1098/rspb.2002.2179 PMC1691180 12495482

[31] Harvey CT, Qureshi SA, MacIsaac HJ 2009 Detection of a colonizing, aquatic, non-indigenous species. Divers. Distrib. 15, 429–437. (doi:10.1111/j.1472-4642.2008.005500.x)

[32] Perry JN 1996 Simulating spatial patterns of counts in agriculture and ecology. Comput. Elect. Agri. 15, 93–109. (doi:10.1016/0168-1699(96)00005-1)

[33] Turner SJ 1993 Soil sampling to detect potato cyst-nematodes (*Globodera* spp.). Ann. Appl. Biol. 123, 349–357. (doi:10.1111/j.1744-7348.1993.tb04097.x)

[34] Lindström T, Håkansson N, Wennergren U 2011 The shape of the spatial kernel and its implications for biological invasions in patchy environments. Proc. R. Soc. B 278, 1564–1571. (doi:10.1098/rspb.2010.1902) 10.1098/rspb.2010.1902 PMC3081748 21047854

[35] Renton M, Shackelford N, Standish RJ 2012 Habitat restoration will help some functional plant types persist under climate change in fragmented landscapes. Glob. Change Biol. 18, 2057–2070. (doi:10.1111/j.1365-2486.2012.0.2677.x)

[36] Renton M, Shackelford N, Standish R 2014 How will climate variability interact with long-term climate change to affect the persistance of plant species in fragmented landscapes? Environ. Conserv. 41, 110–121. (doi:10.1017/S0376892913000490)

[37] Gaston KJ 1994 What is rarity? Dordrecht, The Netherlands: Springer.

[38] Coutts S, van Klinken R, Yokomizo H, Buckley Y 2011 What are the key drivers of spread in invasive plants: dispersal, demography or landscape: and how can we use this knowledge to aid management? Biol. Invas. 13, 1649–1661. (doi:10.1007/s10530-010-9922-5)

[39] With KA 2002 The landscape ecology of invasive spread. Conserv. Biol. 16, 1192–1203. (doi:10.1046/j.1523-1739.2002.01064.x)

[40] Parnell S, Gottwald T, Van Den Bosch F, Gilligan C 2009 Optimal strategies for the eradication of asiatic citrus canker in heterogeneous host landscapes. Phytopathology 99, 1370–1376. (doi:10.1094/PHYTO-99-12-1370) 19900003 10.1094/PHYTO-99-12-1370

[41] Epanchin-Niell RS, Haight RG, Berec L, Kean JM, Liebhold AM, Regan H 2012 Optimal surveillance and eradication of invasive species in heterogeneous landscapes. Ecol. Lett. 15, 803–812. (doi:10.1111/j.1461-0248.2012.01800.x) 22642613 10.1111/j.1461-0248.2012.01800.x

