# Supplementary figures and images for: Do an invasive organism's dispersal characteristics affect how we should search for it?
Source: R Soc Open Sci. 2018 Mar 21;5(3):171784. doi: 10.1098/rsos.171784 (PMC5882706; doi:10.1098/rsos.171784)

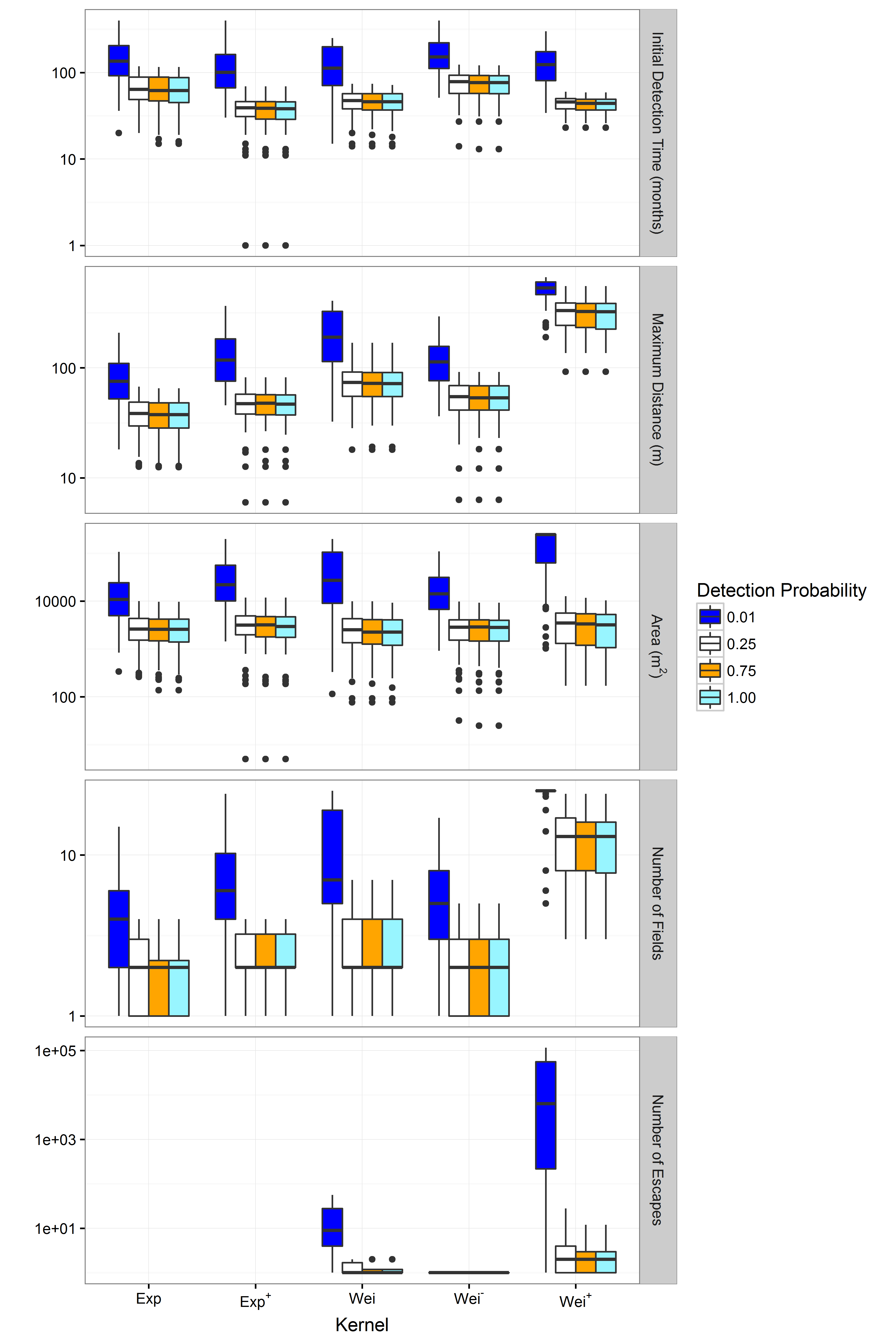

Supplement: Detection probability output [file rsos171784supp1.png]
