# Supplementary material for: Do an invasive organism's dispersal characteristics affect how we should search for it?
Source: R Soc Open Sci. 2018 Mar 21;5(3):171784. doi: 10.1098/rsos.171784 (PMC5882706; doi:10.1098/rsos.171784)
Supplement: Supplementary file 2 [file rsos171784supp2.docx]

Kernel	Density	Mean	SE	Frequency	Mean	SE	Arrangement	Mean	SE	Detection Probability	Mean	SE
**Wei**	0.5	1.29	0.28	1	0.14	0.05	Strategic	0.09	0.03	0.01	10.64	1.52
	1	0.25	0.07	2	0.14	0.04	Grid	0.14	0.05	0.25	0.13	0.04
	2	0.14	0.05	3	0.13	0.04	Random	0.52	0.13	0.75	0.10	0.04
	4	0.07	0.03	4	0.17	0.05				1.00	0.10	0.04
	9	0.04	0.02	6	0.20	0.06						
	16	0.03	0.02	8	0.28	0.07						
	25	0.01	0.01	12	0.23	0.06						
**Wei-**	0.5	0.07	0.03	1	0.01	0.01	Strategic	0.00	0.00	0.01	0.06	0.02
	1	0.02	0.01	2	0.01	0.01	Grid	0.01	0.01	0.25	0.00	0.00
	2	0.01	0.01	3	0.01	0.01	Random	0.06	0.03	0.75	0.00	0.00
	4	0.00	0.00	4	0.01	0.01				1.00	0.00	0.00
	9	0.00	0.00	6	0.01	0.01						
	16	0.00	0.00	8	0.01	0.01						
	25	0.00	0.00	12	0.02	0.01						
**Wei+**	0.5	140.53	20.36	1	23.70	3.33	Strategic	40.65	4.31	0.01	23866.22	2941.27
	1	55.51	6.90	2	26.55	3.72	Grid	23.70	3.33	0.25	2.06	0.45
	2	23.70	3.33	3	33.62	5.02	Random	45.19	8.03	0.75	1.09	0.20
	4	11.38	1.48	4	33.93	4.78				1.00	1.04	0.20
	9	5.06	1.00	6	51.51	8.05						
	16	2.18	0.40	8	73.01	17.14						
	25	1.33	0.22	12	120.87	24.19						
